# Supplementary material for: Enolase inhibitors as therapeutic leads for Naegleria fowleri infection
Source: PLoS Pathog. 2024 Aug 1;20(8):e1012412. doi: 10.1371/journal.ppat.1012412 (PMC11321563; doi:10.1371/journal.ppat.1012412)
Supplement: S1 Text — (DOCX) [file ppat.1012412.s001.docx]

**S1 Text**

**Materials and Methods**

**Protein purification, crystallization, and structure determination**

An *E.coli* codon optimized construct in the vector pQE-30 was purchased from Twist Bioscience containing residues 2-512 of the full length 512 amino acid protein (NF0118810, AmoebaDB, https://amoebadb.org/amoeba/app/). Sequence comparison and secondary structure predictions using Xtalpred (https://xtalpred.godziklab.org/XtalPred-cgi/xtal.pl) were used to design multiple constructs to test for optimal crystallization construct. Five constructs composed of residues 14-512, 28-512, 44-512, 62-512, 80-512 were processed using the SSGCID high throughput pipeline (*20-22*). The constructs were cloned using into the ligation independent cloning (LIC) (*37*) expression vector pBG1861 (*21*) encoding a non-cleavable 6xHis fusion N-terminal tag. Protein was expressed in *E. coli* BL21(DE3)R3 Rosetta cells and two constructs yielded soluble protein, constructs bearing residues *Nf*ENO(44-512) and *Nf*ENO(62-512). These lines were used for large scale (2L) protein production in auto-induction media (*23*) in a LEX Bioreactor (Epiphyte Three Inc.), as previously described (*22*). The expression clones NafoA.00379.a.B4.GE44253 (NfENO(62-512) and NafoA.00379.a.B5.GE44254(NfENO(44-512) are available at <https://www.ssgcid.org/available-materials/ssgcid-proteins/>.

*Nf*ENO(44-512) and *Nf*ENO(62-512) were purified in a two-step protocol consisting of Ni^2+^-affinity and size-exclusion chromatography (SEC). All chromatography was performed on an ÄKTApurifier 10 (GE Healthcare) according to previously described procedures (*20*). Thawed bacterial pellets were lysed by sonication in lysis buffer (25 mM HEPES pH 7.0, 500 mM NaCl, 5% glycerol , 0.5% CHAPS, 30 mM Imidazole, 10 mM MgCl_2_, 1 mM TCEP, 250 ug/ml AEBSF, and 0.025% sodium azide). After sonication, the crude lysate was clarified by mixing with 500 units of Benzonase (RT, 45 min). The lysate was clarified by centrifugation and then passed over a Ni-NTA His-Trap FF 5 ml column (GE Healthcare) which was pre-equilibrated with loading buffer (25 mM HEPES pH 7.0, 500 mM NaCl, 5% glycerol , 30 mM imidazole, 1 mM TCEP, and 0.025% sodium azide). The column was washed and protein pooled and concentrated prior to loading on a SEC column (Superdex 75, GE Healthcare) equilibrated with running buffer (loading buffer without imidazole). The SEC peak fractions eluted as a single large peak in the molecular-mass range ~50 kDa, suggesting monomeric enzyme which was consistent with the size of the denatured purified protein, as determined by SDS-PAGE. Peak fractions were pooled and concentrated using an Amicon purification system (Millipore) to a final concentration of to 73 mg/ml *Nf*ENO(44-512) and 45 mg/ml *Nf*ENO(62-512).

Purified *Nf*ENO(44-512) and *Nf*ENO(62-512) were screened for crystallization in 96-well sitting-drop plates against the JCSG++ HTS (Jena Bioscience) and PACT premier HT96 (Molecular Dimensions) crystal screens. Proteins were diluted in SEC buffer 1:1 to final concentrations 36 mg/ml *Nf*ENO(44-512) and 23 mg/ml *Nf*ENO(62-512). Equal volumes of protein and precipitant solutions were set up at 289 K against reservoir in sitting-drop vapor-diffusion format. In addition to protein-only crystallizations, co-crystallization trials with 2-phosphoglycerate (2-PG, 5 mM) and HEX (5 mM) were also pursued. Only NfENO(44-512) in complex with 2-PG crystallized to form diffraction quality crystals in 85 mM Tris/HCl pH 8.5, 25.5% (w/v) PEG4000, 15% glycerol, and 170 mM sodium acetate, 5 mM 2-PG. The crystals were flash-frozen by plunging directly into liquid nitrogen without cryoprotectant exchange.

X-ray data were collected at 100°K on beamline 21-ID-F, LS-CAT, at the Advanced Photon Source, Argonne National Laboratory. Data were processed with XDS reduced with XSCALE (*24*) and the structure was solved by molecular replacement using the structure of *B. subtilus* ENO (PDB 4A3R) as a search model (*25*). Structures were refined using iterative cycles of Phenix (*26*) followed by manual rebuilding of the structure using Coot (*27*) to yield a resolution of 1.95Å, with the quality of all structures checked using MolProbity (*28*). All data-reduction and refinement statistics are shown in Tables S4 and S5. Structure figures were prepared and analyzed using PyMOL (v.1.5; Schrodinger) and coordinates and structure factors have been deposited with the Protein Data Bank [www.rcsb.org](http://www.rcsb.org) (*29*) with accession number 7UGH.
