# Supplementary material for: Enolase inhibitors as therapeutic leads for Naegleria fowleri infection
Source: PLoS Pathog. 2024 Aug 1;20(8):e1012412. doi: 10.1371/journal.ppat.1012412 (PMC11321563; doi:10.1371/journal.ppat.1012412)
Supplement: S1 Fig — The compounds (1-hydroxy-2-oxopyrrolidin-3-yl) phosphonic acid (deoxy-SF2312) 1, (1-hydroxy-2-oxopiperidin-3-yl) phosphonic acid (HEX) 2, and (1-hydroxy-2-oxoazepan-3-yl) phosphonic acid (HEPTA) 3, were synthesized following published procedures reported in the literature (5, 8). Briefly, a mixture of O-benzylhydroxylamine hydrochloride and chloroformate in pyridine was stirred at room temperature under N2 for 4 h to yield ethyl benzyloxycarbamate (4). Triethyl phosphite in excess of the appropriate dibromoalkane (i.e. n = 1 for Deoxy-SF2312, n = 2 for HEX, and n = 3 for HEPTA) was stirred at 90°C overnight to yield the appropriate alkylbromophoshonate (5) as light yellow oil after purification by silica gel column chromatography (0–8% MeOH in DCM). Potassium carbonate was added to a solution of 4 and 5 in MeCN and the mixture was stirred at 90°C overnight to yield the linear diethyl phosphonate-carbamate (6) as a yellow oil after purification by silica gel column (0–8% MeOH in DCM). Treatment of 6 in THF with LiHMDS at 0°C for 3 h under N2 afforded the cyclized diethyl phosphonate (7) as a yellow oil after purification by silica gel column chromatography (0–8% MeOH in DCM). Hydrolysis of 7 with iodotrimethylsilane in DCM at 0–25°C for 4 h under N2 yielded the benzyloxy phosphonic acid (8) as a yellow solid after reverse phase column chromatography (C18 silica gel; 0–25% MeOH in H2O). Finally, the benzyl group was removed by hydrogenolysis of (1.8 mmol, 8) dissolved in 10 mL of H2O/MeOH (1:1 v/v) containing 30 mmol of acetic acid and palladium on carbon (10%, 50 mg) stirred at 65°C for 18–36 h at 1 atm H2 to give the desired products as an off-white oil (1) or light-yellow oil (2 and 3). (DOCX) [file ppat.1012412.s002.docx]

**S1 Fig. Synthetic route for deoxy-SF2312, HEX, and HEPTA.** The compounds (1-hydroxy-2-oxopyrrolidin-3-yl) phosphonic acid (deoxy-SF2312) **1**, (1-hydroxy-2-oxopiperidin-3-yl) phosphonic acid (HEX) **2**, and (1-hydroxy-2-oxoazepan-3-yl) phosphonic acid (HEPTA) **3**, were synthesized following published procedures reported in the literature (*5, 8*). Briefly, a mixture of *O*-benzylhydroxylamine hydrochloride and chloroformate in pyridine was stirred at room temperature under N_2_ for 4 h to yield ethyl benzyloxycarbamate (**4**). Triethyl phosphite in excess of the appropriate dibromoalkane (*i.e*. n = 1 for Deoxy-SF2312, n = 2 for HEX, and n = 3 for HEPTA) was stirred at 90 ˚C overnight to yield the appropriate alkylbromophoshonate (**5**) as light yellow oil after purification by silica gel column chromatography (0–8% MeOH in DCM). Potassium carbonate was added to a solution of **4** and **5** in MeCN and the mixture was stirred at 90 ˚C overnight to yield the linear diethyl phosphonate-carbamate (**6**) as a yellow oil after purification by silica gel column (0–8% MeOH in DCM). Treatment of **6** in THF with LiHMDS at 0 ˚C for 3 h under N_2_ afforded the cyclized diethyl phosphonate (**7**) as a yellow oil after purification by silica gel column chromatography (0–8% MeOH in DCM). Hydrolysis of **7** with iodotrimethylsilane in DCM at 0–25 ˚C for 4 h under N_2_ yielded the benzyloxy phosphonic acid (**8**) as a yellow solid after reverse phase column chromatography (C18 silica gel; 0–25% MeOH in H_2_O). Finally, the benzyl group was removed by hydrogenolysis of (1.8 mmol, **8**) dissolved in 10 mL of H_2_O/MeOH (1:1 v/v) containing 30 mmol of acetic acid and palladium on carbon (10%, 50 mg) stirred at 65 ˚C for 18–36 h at 1 atm H_2_ to give the desired products as an off-white oil (**1**) or light-yellow oil (**2** and **3**).
