# Supplementary material for: Enolase inhibitors as therapeutic leads for Naegleria fowleri infection
Source: PLoS Pathog. 2024 Aug 1;20(8):e1012412. doi: 10.1371/journal.ppat.1012412 (PMC11321563; doi:10.1371/journal.ppat.1012412)
Supplement: S2 Fig — 1H and 13C NMR spectra were obtained using Bruker Advance 500 MHz spectrometers. Chemical shifts are reported in parts per million (ppm). Spectra are referenced to residual solvent peaks. Flash column chromatography was carried out using ZEOCHEM silica gel (40–63 μm). Analytical and preparative thin-layer chromatography (TLC) were performed on Sorbtech silica G TLC plates. All non-aqueous reactions were performed under an inert atmosphere of nitrogen in flame-dried glassware containing a stir bar unless otherwise noted. Acetonitrile (ACN), tetrahydrofuran (THF), dichloromethane (DCM), methanol (MeOH), and pyridine were obtained from commercial sources and dried following standard distillation procedures. All other solvents were obtained from commercial sources and used without drying unless otherwise noted. All water and aqueous solutions were made using deionized (DI) water. (DOCX) [file ppat.1012412.s003.docx]

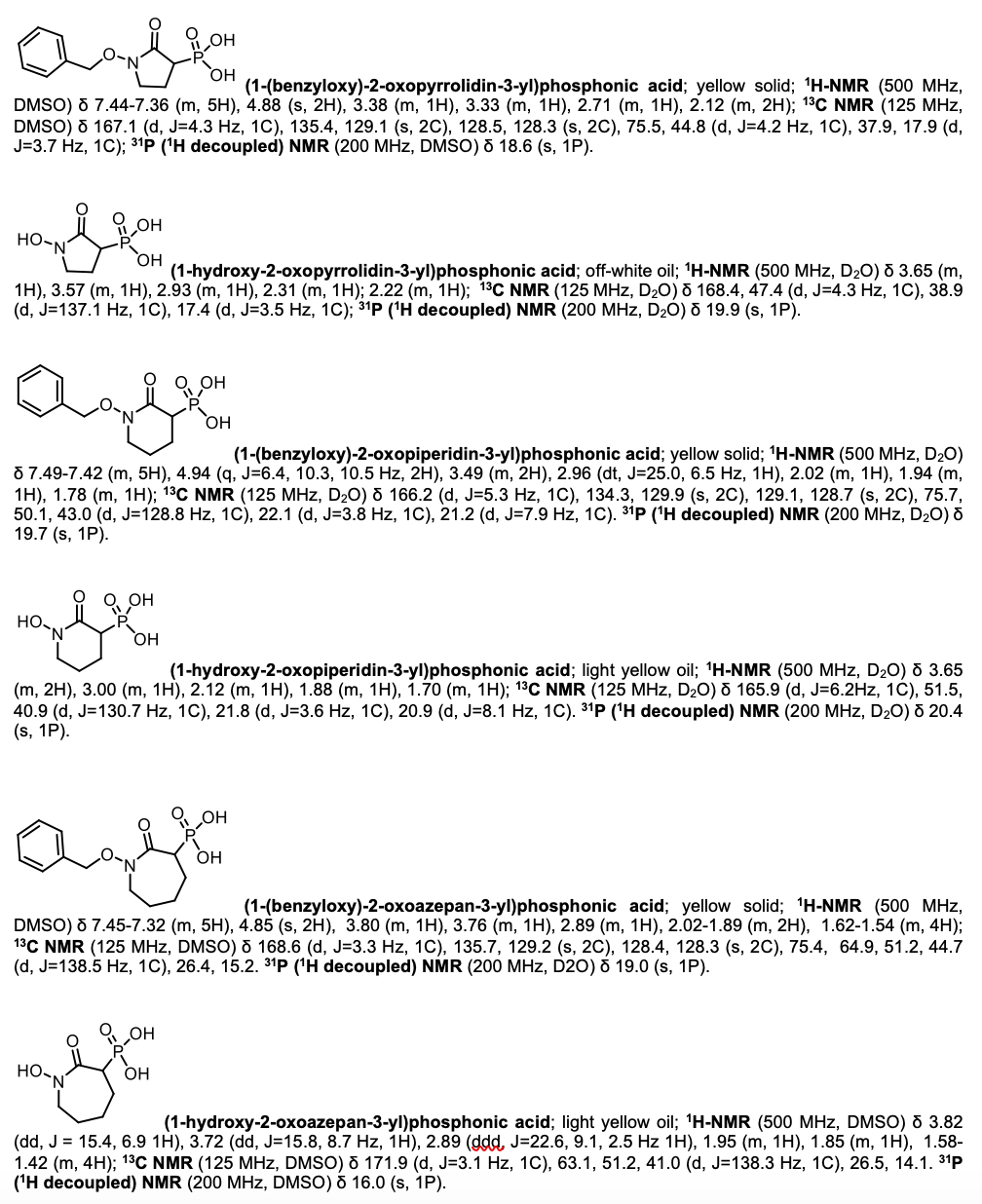


**
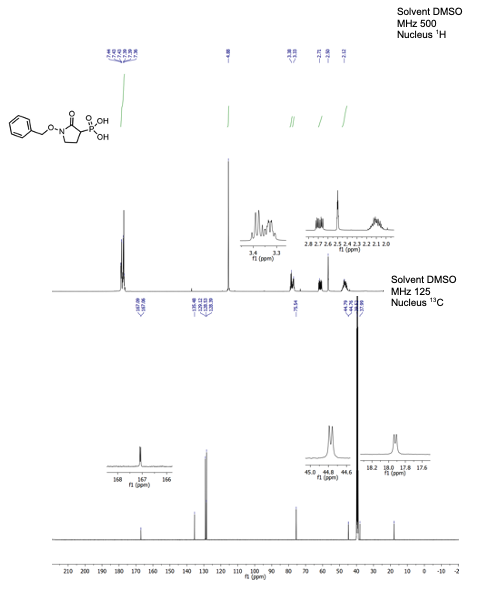
**

**
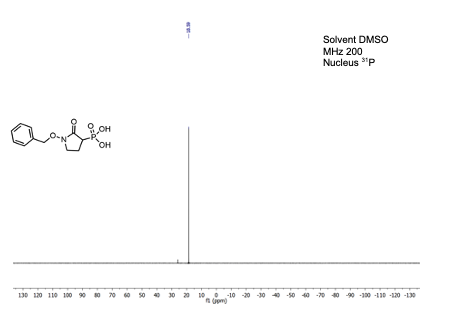
**

**
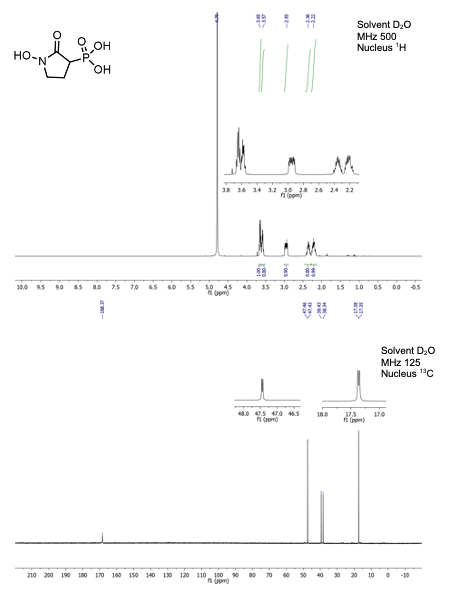
**

**
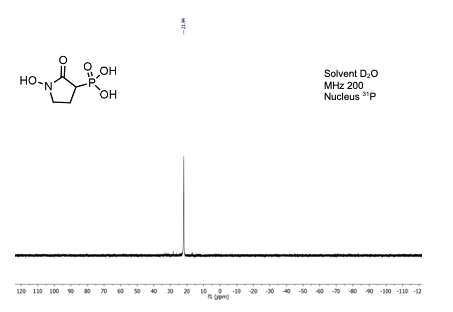
**

**
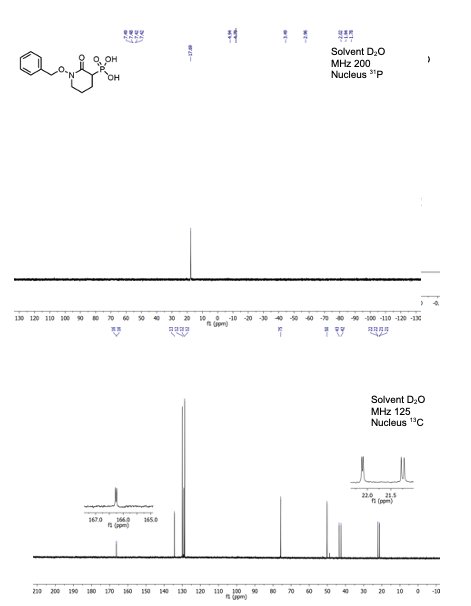
**

**
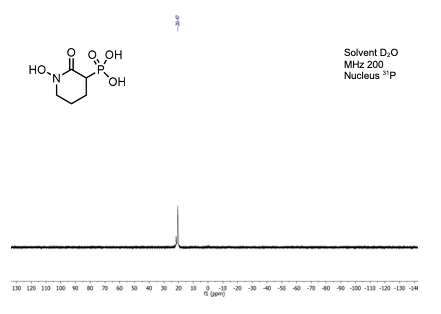
**

**
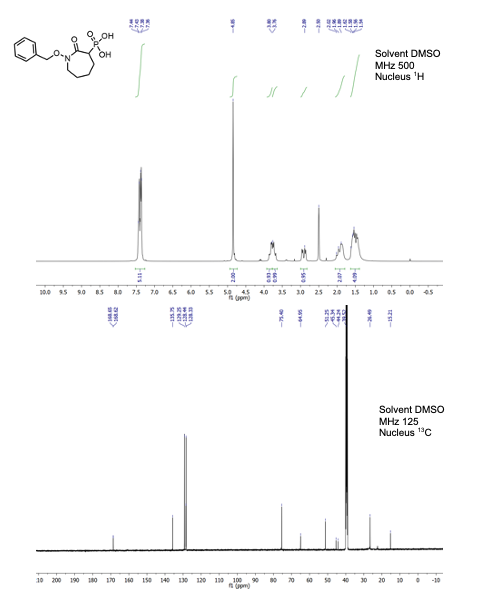
**

**
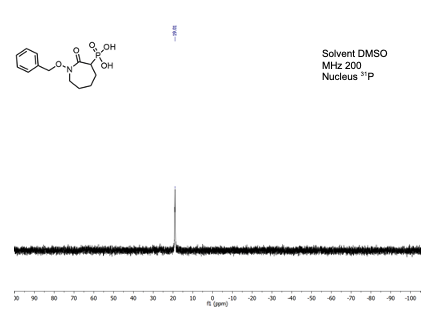
**

**
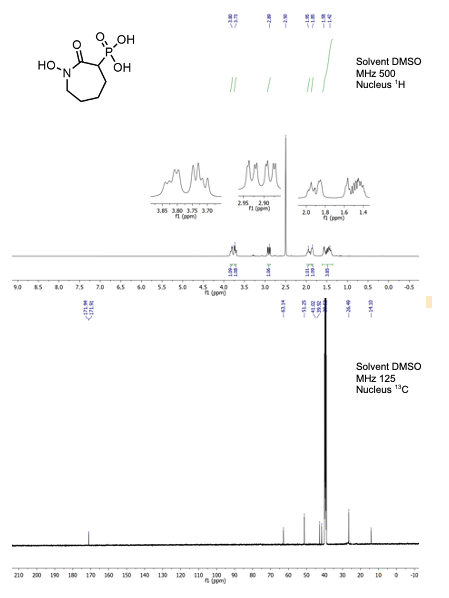
**

**
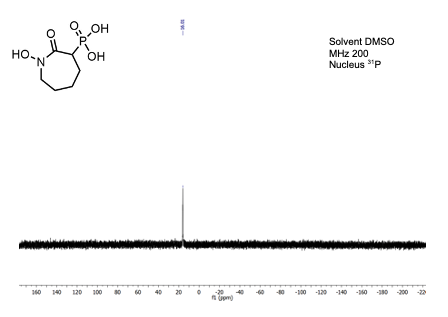
**

**S2 Fig. Spectroscopic characterization of deoxy-SF2312, HEX and HEPTA**. ^1^H and ^13^C NMR spectra were obtained using Bruker Advance 500 MHz spectrometers. Chemical shifts are reported in parts per million (ppm). Spectra are referenced to residual solvent peaks. Flash column chromatography was carried out using ZEOCHEM silica gel (40-63 µm). Analytical and preparative thin-layer chromatography (TLC) were performed on Sorbtech silica G TLC plates. All non-aqueous reactions were performed under an inert atmosphere of nitrogen in flame-dried glassware containing a stir bar unless otherwise noted. Acetonitrile (ACN), tetrahydrofuran (THF), dichloromethane (DCM), methanol (MeOH), and pyridine were obtained from commercial sources and dried following standard distillation procedures. All other solvents were obtained from commercial sources and used without drying unless otherwise noted. All water and aqueous solutions were made using deionized (DI) water.
