# Supplementary material for: Enolase inhibitors as therapeutic leads for Naegleria fowleri infection
Source: PLoS Pathog. 2024 Aug 1;20(8):e1012412. doi: 10.1371/journal.ppat.1012412 (PMC11321563; doi:10.1371/journal.ppat.1012412)
Supplement: S3 Fig — [https://doi.org/10.1093/nar/gku316]. The top lines show the secondary structure elements of NfENO. The scale above the alignment corresponds to the NfENO sequence. Active site residues are highlighted with a blue triangle; the black star marks the position of NfENO Lys243. (DOCX) [file ppat.1012412.s004.docx]

**S3 Fig. ClustalOmega alignment of *Nf*ENO with human ENOs colored by percent equivalent score**. [<https://doi.org/10.1093/nar/gku316>]. The top lines show the secondary structure elements of *Nf*ENO. The scale above the alignment corresponds to the *Nf*ENO sequence. Active site residues are highlighted with a blue triangle; the black star marks the position of *Nf*ENO Lys243.
