# Supplementary material for: Enolase inhibitors as therapeutic leads for Naegleria fowleri infection
Source: PLoS Pathog. 2024 Aug 1;20(8):e1012412. doi: 10.1371/journal.ppat.1012412 (PMC11321563; doi:10.1371/journal.ppat.1012412)
Supplement: S4 Fig — (A) Superposition of the active sites of HEX-bound ENO2 and HEX-docked NfENO. (B) NfENO lacks Arg-372 and likely engages with HEX through Lys-243, which differs from (C) ENO2. (DOCX) [file ppat.1012412.s005.docx]

**
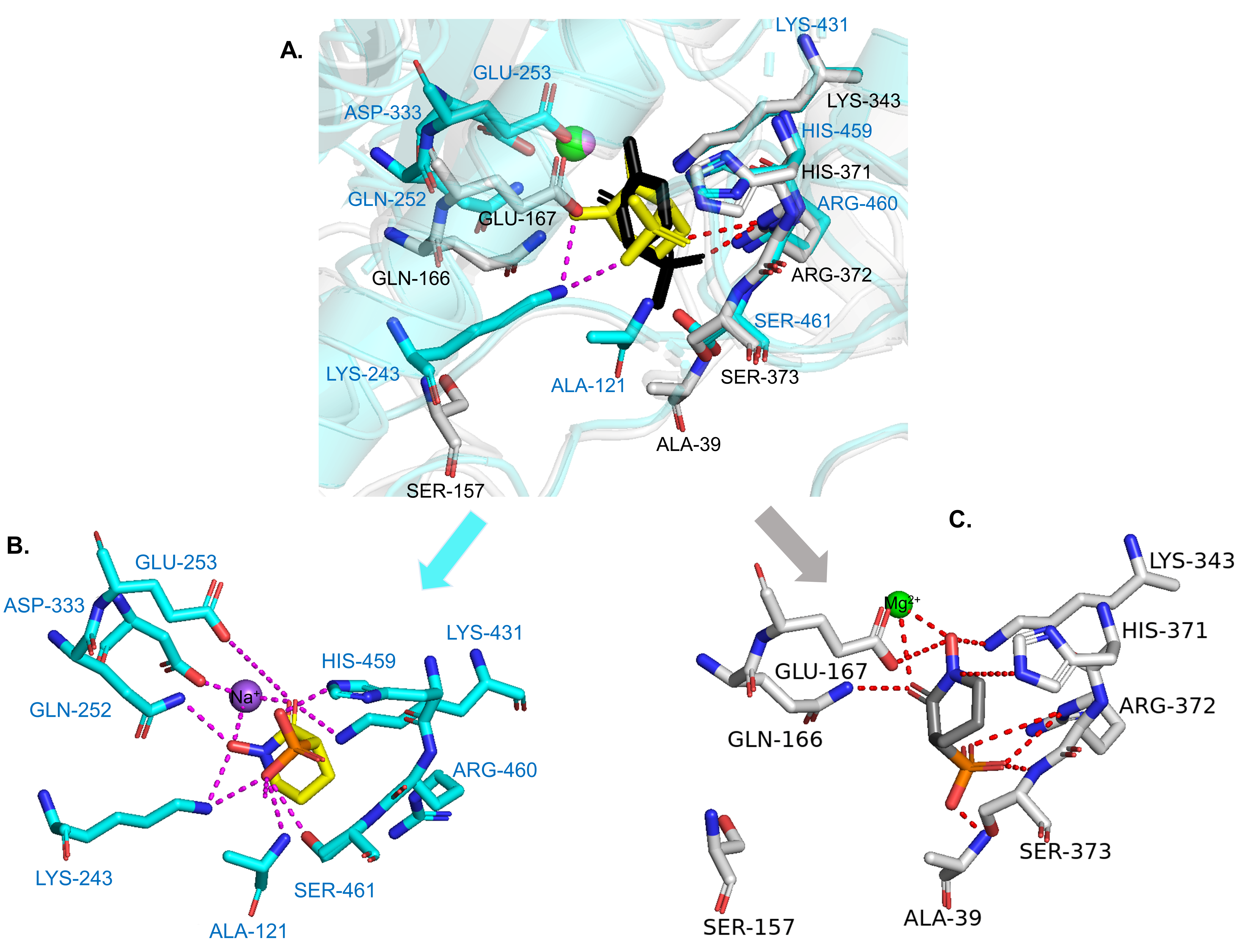
**

**S4 Fig.**  **Comparison of the active sites of HEX-bound ENO2 and HEX-docked *Nf*ENO .** (A) Superposition of the active sites of HEX-bound ENO2 and HEX-docked *Nf*ENO. (B) NfENO lacks Arg-372 and likely engages with HEX through Lys-243, which differs from (C) ENO2.
