# Supplementary material for: Enolase inhibitors as therapeutic leads for Naegleria fowleri infection
Source: PLoS Pathog. 2024 Aug 1;20(8):e1012412. doi: 10.1371/journal.ppat.1012412 (PMC11321563; doi:10.1371/journal.ppat.1012412)
Supplement: S5 Fig — Trophozoites were cultured from brains of a HEX-treated (HEX) or PBS-treated (Vehicle) rodent for two weeks in media followed by testing against HEX in a standard viability assay. Both cultures responded similarly, with EC50 values of 0.09 ± 0.04 and 0.1 ± 0.08 μM for HEX- or Vehicle-treated rodents, respectively. Drug concentrations were tested in triplicate, with some error bars smaller than the graphing symbols. (DOCX) [file ppat.1012412.s006.docx]

**S5 Fig.**  **Amoeba resistance to HEX does not explain the therapeutic failure observed in the one HEX-treated rodent that succumb to infection.** Trophozoites were cultured from brains of a HEX-treated (HEX) or PBS-treated (Vehicle) rodent for two weeks in media followed by testing against HEX in a standard viability assay. Both cultures responded similarly, with EC_50_ values of 0.09 ± 0.04 and 0.1 ± 0.08 µM for HEX- or Vehicle-treated rodents, respectively. Drug concentrations were tested in triplicate, with some error bars smaller than the graphing symbols.
