# Supplementary material for: Enolase inhibitors as therapeutic leads for Naegleria fowleri infection
Source: PLoS Pathog. 2024 Aug 1;20(8):e1012412. doi: 10.1371/journal.ppat.1012412 (PMC11321563; doi:10.1371/journal.ppat.1012412)
Supplement: S1 Table — (DOCX) [file ppat.1012412.s007.docx]

**S1 Table. NfENO sequences from Uniprot, AmoebaDB, and the truncation used for crystallography that is deposited in the PDB.**

| **Source** | **ID** | **Sequence** |
| --- | --- | --- |
| Uniprot | tr\|A0A6A5BXC3\|A0A6A5BXC3_NAEFO | MTDKQPVSEYLQNHNLQKLVEDALNECYNANASDPVGFLGHFFLNRGKKGAVNRVDKLVGREILDSRGNPTVEVDVYANGQKRPVATASAPSGASTGSNEAHELRDGDKSRYLGKGVLKAVKNVNDVLGKAVEGKSLENLTELDQALIDADGDELKSNLGGNAITACSFALATAGAAVRNEELFLYLARAFHGADKFENLKFRLPTPMVNILNGGKHAGGRLQIQEFMILPKENQPFREK  VRCVAEVYQHLGKILAERAGPSAKNVGDEGGFAPNLETADEALNYIEEAIGKAGYKVGED  VFLALDAASSEFYNSDTKKYEITQQKEFLTSEEMVEYYVQLVNRHPAIISIEDGLEEKDY  EGWKLLTERLGSKIMLVGDDLYTTNTRLIKQGIEEKWANALLLKVNQIGTITEAMNAARM  IFNVGQKVIVSHRSGETATTLISDLVVGIGATHIKTGATARGERVSKYNRLLQIEEYLEQ  HGLLA |
| AmoebaDB | NF0118810 | MLYLVLMLVENGFGQNCFLHSQKICSNMTDKQPVSEYLQNHNL**Q**KLVEDALNECYNANASDPVGFLGHFFLNRGKKGAVNRVDKLVGREILDSRGNPTVEVDVYANGQKRPVATASAPSGASTGSNEAHELRDGDKSRYLGKGVLKAVKNVNDVLGKAVEGKSLENLTELDQALIDADGDELKSNLGGNAITACSFALATAGAAVRNEELFLYLARAFHGADKFENLKFRLPTPMVNILNGGKHAGGRLQIQEFMILPKENQPFREKVRCVAEVYQHLGKILAERAGPSAKNVGDEGGFAPNLETADEALNYIEEAIGKAGYKVGEDVFLALDAASSEFYNSDTKKYEITQQKEFLTSEEMVEYYVQLVNRHPAIISIEDGLEEKDYEGWKLLTERLGSKIMLVGDDLYTTNTRLIKQGIEEKWANALLLKVNQIGTITEAMNAARMIFNVGQKVIVSHRSGETATTLISDLVVGIGATHIKTGATARGERVSKYNRLLQIEEYLEQHGLLA |
| PDB (truncation used for crystallography) | 7UGH_1 | MAHHHHHHQKLVEDALNECYNANASDPVGFLGHFFLNRGKKGAVNRVDKLVGREILDSRGNPTVEVDVYANGQKRPVATASAPSGASTGSNEAHELRDGDKSRYLGKGVLKAVKNVNDVLGKAVEGKSLENLTELDQALIDADGDELKSNLGGNAITACSFALATAGAAVRNEELFLYLARAFHGADKFENLKFRLPTPMVNILNGGKHAGGRLQIQEFMILPKENQPFREKVRCVAEVYQHLGKILAERAGPSAKNVGDEGGFAPNLETADEALNYIEEAIGKAGYKVGEDVFLALDAASSEFYNSDTKKYEITQQKEFLTSEEMVEYYVQLVNRHPAIISIEDGLEEKDYEGWKLLTERLGSKIMLVGDDLYTTNTRLIKQGIEEKWANALLLKVNQIGTITEAMNAARMIFNVGQKVIVSHRSGETATTLISDLVVGIGATHIKTGATARGERVSKYNRLLQIEEYLEQHGLLA |
