# Supplementary material for: Enolase inhibitors as therapeutic leads for Naegleria fowleri infection
Source: PLoS Pathog. 2024 Aug 1;20(8):e1012412. doi: 10.1371/journal.ppat.1012412 (PMC11321563; doi:10.1371/journal.ppat.1012412)
Supplement: S2 Table — Values for the outer resolution shell are given in parentheses. (DOCX) [file ppat.1012412.s008.docx]

**S2 Table. X-ray Data collection and processing.** Values for the outer resolution shell are given in parentheses.
