# Supplementary material for: Enolase inhibitors as therapeutic leads for Naegleria fowleri infection
Source: PLoS Pathog. 2024 Aug 1;20(8):e1012412. doi: 10.1371/journal.ppat.1012412 (PMC11321563; doi:10.1371/journal.ppat.1012412)
Supplement: S3 Table — Values for the outer shell are given in parentheses. (DOCX) [file ppat.1012412.s009.docx]

**S3 Table. Crystallographic structure solution and refinement quality statistics**. Values for the outer shell are given in parentheses.
