# Supplementary material for: Enolase inhibitors as therapeutic leads for Naegleria fowleri infection
Source: PLoS Pathog. 2024 Aug 1;20(8):e1012412. doi: 10.1371/journal.ppat.1012412 (PMC11321563; doi:10.1371/journal.ppat.1012412)
Supplement: S4 Table — Fold-change (FC) is the mean ratio of peak area of a metabolite detected in the first group divided by the corresponding value in the second group. One-way ANOVA with the Tukey method for post-test was used for metabolite abundance significance test, and the Benjamini and Hochberg method was used for multiple test correction. ND stands for not detected. The metabolite was either not present in the sample or its concentration in sample is smaller than the sensitivity of the analytical platform. (DOCX) [file ppat.1012412.s010.docx]

**S4 Table. Unbiased metabolomics results for amoebae grown in the presence of HEX.** Fold-change (FC) is the mean ratio of peak area of a metabolite detected in the first group divided by the corresponding value in the second group. One-way ANOVA with the Tukey method for post-test was used for metabolite abundance significance test, and the Benjamini and Hochberg method was used for multiple test correction. ND stands for not detected. The metabolite was either not present in the sample or its concentration in sample is smaller than the sensitivity of the analytical platform.
