# Supplementary material for: Enolase inhibitors as therapeutic leads for Naegleria fowleri infection
Source: PLoS Pathog. 2024 Aug 1;20(8):e1012412. doi: 10.1371/journal.ppat.1012412 (PMC11321563; doi:10.1371/journal.ppat.1012412)
Supplement: S5 Table — 1Relative to average intensities of metabolites detected in cells grown under standard (+glc) conditions. (DOCX) [file ppat.1012412.s011.docx]

**S5 Table**. **Fold-change in abundance^1^ of metabolites in response to carbon source and HEX treatment, relative to growth in glucose alone.** ^1^Relative to average intensities of metabolites detected in cells grown under standard (+glc) conditions.

| Metabolite | +HEX | -glc/+gly | -glc/+gly +HEX |
| --- | --- | --- | --- |
| Glucose | 0.51 | 0.41 | 0.41 |
| G6P | 7.4 | 0.95 | 13 |
| F6P | 4.5 | 0.83 | 6.5 |
| Gly3P | 17.9 | 1.5 x 10^2^ | 5.1 x 10^2^ |
| 2-/3-PG | 78 | 11 | 3.7 x 10^2^ |
| PYR | 1.2 | 5.8 | 4.3 |
