# Supplementary material for: Enolase inhibitors as therapeutic leads for Naegleria fowleri infection
Source: PLoS Pathog. 2024 Aug 1;20(8):e1012412. doi: 10.1371/journal.ppat.1012412 (PMC11321563; doi:10.1371/journal.ppat.1012412)
Supplement: S6 Table — 1Relative to average intensities of metabolites detected in cells grown in -glc/+gly conditions. (DOCX) [file ppat.1012412.s012.docx]

**S6 Table.** **Fold-change in abundance of metabolites of cells grown in glycerol after HEX treatment,** ^1^**relative to growth in glycerol alone.** ^1^Relative to average intensities of metabolites detected in cells grown in -glc/+gly conditions.

| Metabolite | -glc/+gly +HEX |
| --- | --- |
| Glucose | 1.2 |
| G6P | 2.9 |
| F6P | 1.7 |
| Gly3P | 26 |
| 2-/3-PG | 7.2 |
| PYR | 0.58 |
